# Experience of Patients with COPD of Pharmacists’ Provided Care: A Qualitative Study

**DOI:** 10.3390/pharmacy9030119

**Published:** 2021-06-29

**Authors:** Omowumi Idowu, Tatiana Makhinova, Maira Quintanilha, Nese Yuksel, Theresa J. Schindel, Ross T. Tsuyuki

**Affiliations:** 1 Faculty of Pharmacy and Pharmaceutical Sciences, University of Alberta, Edmonton, AB T6G 1C9, Canada; omowumi@ualberta.ca (O.I.); nese.yuksel@ualberta.ca (N.Y.); terri.schindel@ualberta.ca (T.J.S.); 2 Quali Q Inc.: Qualitative Research Mentoring and Consulting, Edmonton, AB T6H 5Y7, Canada; maira@ualberta.ca; 3 Department of Pharmacology, Faculty of Medicine and Dentistry, University of Alberta, Edmonton, AB T6G 1C9, Canada; rtsuyuki@ualberta.ca

**Keywords:** COPD, pharmacists, qualitative research, patient experience, patient–pharmacist relationship, qualitative description, patient-centred care

## Abstract

Chronic obstructive pulmonary disease (COPD) is associated with high global morbidity and mortality. Pharmacists are uniquely positioned to provide services which may reduce the burden of this disease on the health system, patients, and their families. The study aimed to understand the perceptions and experiences of patients living with COPD with pharmacists’ provided care in COPD diagnosis and management. The study was guided by qualitative description methodology and reported using the consolidated criteria for reporting qualitative research (COREQ) checklist. We conducted semi-structured interviews with 12 participants who were recruited from community pharmacies, seniors’ centres, a general practice clinic, and a pulmonary rehabilitation centre. Using qualitative content analysis, we identified categories that revealed great variation in participants’ experience of pharmacy care based on the depth of patient–pharmacist engagement. Participants who regarded their pharmacists as an essential member of their healthcare team and those who did not, had contrasting experiences with education, communication, and ability to form connections with their pharmacists. For patients with COPD, it is important that the pharmacist is proactive in engaging patients through effective communication, education/provision of relevant information, identification of patient needs, and consistent provision of care with empathy.

## 1. Introduction

Chronic obstructive pulmonary disease (COPD) is currently the fourth leading cause of death in Canada, affecting about 4% of the population [1]. COPD, under which older terms such as chronic bronchitis and emphysema fall, is characterised by progressive lung function decline. Though currently incurable, COPD is preventable and treatable and is mostly caused by tobacco smoking with characteristic symptoms of dyspnea and cough, with or without sputum production [2,3]. Despite COPD being a leading cause of global death already, its contribution to global mortality is projected to increase in the years ahead due to increased exposure to risk factors, an ageing population, and low awareness of the disease by individuals [4,5,6]. Additionally, misdiagnosis and underdiagnosis is a significant gap in care, which further increase the burden of COPD since patients with undiagnosed COPD experience exacerbation-like events as frequently as those with confirmed diagnosis [7].

Collaboration between healthcare team members, including community pharmacists, and COPD patients (with their caregivers) is necessary for optimal disease management. Community pharmacists can be instrumental in COPD care optimisation as their scope of practice has evolved over the years from the traditional dispensing roles to patient-centred care. The Institute of Medicine defines patient-centred care as ‘care that is respectful of, and responsive to individual patient preferences, needs, and values and ensuring that patient values guide all clinical decisions’ [8]. Patient preferences and expectations are key determinants for producing patient-centred care [9]. Community pharmacists are easily accessible and perceived to be affordable by the public, making them often the first point of contact in the healthcare system [10,11,12]. Pharmacists can play crucial roles in all the key stages of patients’ pathways through targeted disease screening with risk assessment, by providing education on disease awareness and risk prevention, and by optimising therapy management and supporting patients with self-care plans, monitoring, and transition of care [10,13]. Patient education and risk prevention are primary prevention strategies which reduce the incidence and prevalence of diseases. Through positive changes in the COPD pathway, pharmacists have been shown to improve therapeutic, safety, and humanistic outcomes, as well as contribute to more cost-effective healthcare [14]. The provision of direct patient care by pharmacists has also been shown to have beneficial effects across various disease states, healthcare settings, and patient outcomes [14,15,16,17,18]. All these factors increase the potential of pharmacists to be effective healthcare team members and contribute to COPD patients’ care involving case-finding, clinical assessment, optimal pharmacotherapy, referrals, and supporting self-management.

To achieve and enhance the quality of patient-centred care which produces more positive outcomes in patient management, it is important to understand patients’ experiences [19]. With regard to gaps in care, some patients with COPD are documented to have experienced substandard care service delivery characterised by poor accessibility to healthcare services, lack of information and capability to make informed decisions, and poor relationships with healthcare providers [20,21]. The most vulnerable groups of respondents were solitary and had restricted financial and social support [20]. When characterising the relationship with healthcare providers, patients often judged a successful relationship with their physicians based on the level of empathy and support provided to the patients during the disease management process [20]. Importantly, patients’ negative perception of their physicians contributes to poor adherence to treatment and failure to modify health behaviors. Additionally, poor patient–physician relationships may influence a patients’ choice not to seek medical advice when needed, thus negatively impacting their quality of life and disease-coping mechanisms [20].

Since interprofessional care is critical in COPD management, it is important to know what patients think about the roles of other healthcare team members, specifically pharmacists [22]. In the management of other chronic conditions, such as asthma, diabetes, cystic fibrosis, and hypertension, patients perceived the pharmacist as a trusted information provider, care provider, or adviser. Patients were comfortable speaking with the pharmacist about their health and appreciated their pharmacists’ role in providing care. Overall, patients had a positive perception of pharmacists’ care [23,24,25,26]. With respect to COPD, however, only the general experiences of patients and interactions with physicians have been explored, leading to a need to explore these patients’ unique experiences of pharmacists’ provided care. This will enable us to understand the experiences of patients with COPD of pharmacist-provided care and inform on how pharmacist-provided care in COPD diagnosis and management can be improved from the patients’ perspectives. With the intention of informing an improvement of COPD care by pharmacists, the aim of the study was to understand the perceptions and experiences of patients living with COPD of pharmacists’ care in COPD diagnosis and management.

## 2. Materials and Methods

### 2.1. Study Design

Semi-structured, one-on-one interviews were conducted with patients with COPD to enable us to understand their perceptions of pharmacists and their experiences of pharmacists’ care. The interviews were guided methodologically by qualitative description (QD), which is aimed at understanding and describing a phenomenon through the views of people who have experienced such phenomenon in their natural setting [27,28]. QD is embedded in the naturalistic approach, enabling an understanding of the phenomenon of interest through the meanings participants attach to them. The ontological position in this methodology is relativism, where reality is subjective and varies among individuals. The epistemological assumption is subjectivism, which accepts that multiple interpretations of reality can co-exist. The goal of QD is to describe the phenomenon in a rich and easily understandable manner [29].

### 2.2. Participants: Sampling and Recruitment

Adults (aged 18 years and above) with a diagnosis of COPD, residing in the Edmonton area were invited to join the study through recruitment posters in 11 community pharmacies, two seniors’ centres, two pulmonary function laboratories, two hospitals, one general practice clinic, and one pulmonary rehabilitation center. Information about the study, including the study eligibility criteria, the expectations from participants, the incentive for participation in the study (USD 25 gift card), and the contact details (phone, email) of two members of the research team was disseminated via recruitment posters. The incentive value of USD 25 was considered appropriate to recognise the participants’ involvement without being considered an inducement to participate in the study. Four practicing community pharmacists were contacted inviting them to discuss the study with their eligible patients. A short presentation was provided for members of a respiratory club at the pulmonary rehabilitation centre to increase the awareness of patients with COPD about the study. Of all these places where our study was advertised, only those from the pulmonary clinic and the community pharmacies contacted the research team. Of the 14 individuals that were recruited, one declined participation after being informed about the study objectives, while another could not be interviewed due to the global pandemic at the time of the study.

Within the settings purposefully selected for recruitment, participants were recruited through convenience sampling. Criteria for study participation were a diagnosis of COPD, experience with pharmacist-provided care, and ability to communicate in English and give informed consent. The first six participants were from a pulmonary rehabilitation centre. Three participants were recruited from a community pharmacy where the pharmacist is a certified respiratory educator (CRE), and the three others were from three pharmacies where the pharmacists are not CREs. Interviews were conducted until data saturation was reached. Data saturation was defined as the non-emergence of new data in response to interview questions or new themes and codes after analysis of all collected data [30]. Two members of the research team agreed that data saturation was reached when the data from the last two interviews did not yield any new categories different from the previously analysed.

### 2.3. Data Collection

Data were collected between October 2019 and January 2020 through digital audio recordings of the interviews. In 7 of the interviews, there were 2 researchers present (OI and TM or MQ) after which OI conducted the remaining 5 interviews alone. The presence of other researchers at the interviews enhanced the quality of notes taken and data interpretation later on in the process. Moreover, field notes taken during and after the interviews enriched the data analysis.

Interview lengths ranged between 20 and 114 min, with the average being 54 min. The variation in length reflected interactions with the participants. The longest interview involved extensive discussion with the participant. In situations where participants experienced shortness of breath, the interviewers focused on probing and follow-up questions. Before the interviews started, research information was reviewed with the participants, and a copy of the information sheet was provided for their documentation. Participants were asked if there were any questions after which their written consent to participate in the audio-recorded interview was obtained. Interviews were conducted at locations chosen by participants. Thus, 9 interviews were conducted at the participants’ homes, two were conducted at a meeting room at the University of Alberta, and one was conducted at the participant’s pulmonary rehabilitation centre. In one interview, a non-participant (participant’s spouse) was present. However, her consent to participate in the study was not obtained in writing. Thus, her discussions are excluded from our findings.

The interview guide (Appendix A) was developed based on the phenomenon of interest and drawn from the review of studies that had explored the experiences of patients with pharmacists’ provided care in other disease areas. The interview guide was also reviewed and updated during the data collection process. The interview questions covered experiences with COPD diagnosis, management, preventive services such as smoking cessation and vaccination, participants’ perception of their pharmacists’ care, as well as their expectations. Open-ended questions were used to encourage participants to discuss their experiences. There were 5 iterations of the interview guide. Continuous updates of the interview guide enabled the research team to collect as much relevant and specific information in relation to the aim of this study as possible. As an example of the types of changes that were made, the first version of the interview guide started with a question asking participants about their life prior to being diagnosed with COPD to allow the participants to ease into the interview. However, we observed that this question elicited a range of responses that were not crucial to our study objectives. Thus, this question was reviewed and changed to ‘Who helps you manage your COPD?’ to set a context for the interview. Furthermore, in response to ‘Tell me more about your experiences with your pharmacists’ provided care’, some participants’ response was centred on medication dispensing. Thus, in those interviews, we included ‘What role did your pharmacist play?’ to enable participants to reflect on their interactions with the pharmacist and thereby share their experiences. Appendix A is the final version of the interview guide. Additionally, a questionnaire was administered to collect demographic data such as age, sex, time of diagnosis, presence of co-morbidities, frequency of pharmacy visits, and how many times the participants had exacerbations within the previous 12 months (Appendix B).

### 2.4. Data Analysis

The collected data were analysed using qualitative content analysis [31,32]. As our aim was to describe the phenomenon of interest, qualitative content analysis was an appropriate approach [31]. We stayed close to participants’ own words, which is commonly used in the qualitative description. The phases involved in this approach to analysis are data familiarisation, generation of initial codes, searching, review and naming of codes, and the reporting of the analysed data [33].

The interviews were transcribed by a third-party company and then reviewed by OI for accuracy. Transcribed interviews were independently analysed (OI and MQ), and then two researchers discussed the consistency of the coding process and the codes. A more experienced member of the team (MQ) reviewed three of the transcripts coded by OI to ensure the validity of the codes and categories that were created. Categories were discussed and refined to best capture participants’ experiences. Data analysis was carried out concurrently with data collection. Transcripts were initially read to ensure there were no transcribing errors and then reread to enable immersion. The transcripts were then read line by line and coded, capturing phrases that described participants’ perceptions and experiences. While reflecting on the codes and their meanings, notes were taken to document first impressions. The initial codes came from the transcript text and were used as a guiding scheme for subsequent transcripts. Codes were then sorted by their similarities into categories from which later in the process overarching themes were induced [31]. The data was organised using the NVivo^®^12 software. To provide guidance during the reporting of this study, the consolidated criteria for reporting qualitative research (COREQ) checklist (Appendix C) was used [34]. Table 1 illustrates the coding process.

### 2.5. Trustworthiness and Reflexivity

Trustworthiness is an essential component of high-quality research. Elements of trustworthiness include credibility, transferability, dependability, confirmability, and reflexivity [35,36]. Credibility was ensured through reflective journaling during the research process, peer debriefing during data analysis, and the inclusion of field notes in the iterative coding process. To facilitate transferability, the research context, participants and settings are described in a rich manner. To ensure dependability, two researchers discussed the process of data analysis and codes, making appropriate adjustments as necessary. This is in addition to a detailed reporting of the research process. Confirmability was ensured through a detailed methodological description.

Lamb and Huttlinger defined reflexivity as ‘a self-awareness and an awareness of the relationship between the investigator and the research environment’ [37]. Our research team consisted of three female researchers, two of whom are non-practicing pharmacists (OI and TM), and the last, (MQ), a qualitative researcher. Both OI, who is a master’s student, and TM (an assistant professor) have interests in improving the pharmacy care experience of patients living with COPD. At the time of the interviews, OI had taken a graduate study course on qualitative research methods. MQ has over 11 years of experience as a qualitative researcher. TM has experience in conducting qualitative research. OI conducted the interviews, with support from TM and MQ. Throughout the research process, team members discussed their personal views on participants’ experience of pharmacy care. After every interview, OI and either TM or MQ appraised the interview, the appropriateness of the questions, the interview setting, and discussed the level of comfort of participants in answering the interview questions. Reflexivity was also addressed through journaling during the research process.

## 3. Results

Six men and six women participated in this study. The participants’ age ranged from 46 to 85 years old and had been living with COPD for between 2 to 22 years. The participants recruited through the pulmonary rehabilitation centre were members of the ‘Breathe Easy’ program. The Breathe Easy program is a pulmonary rehabilitation program for people diagnosed with chronic lung disease [38]. All participants accessed care from different pharmacies except for three participants who used the same pharmacy. Table 2 gives an overview of the participants’ characteristics. Out of six participants recruited through community pharmacies, for three of them their pharmacist was a certified respiratory educator (CRE), while for the other three, their pharmacists were not.

**Figure 1:**
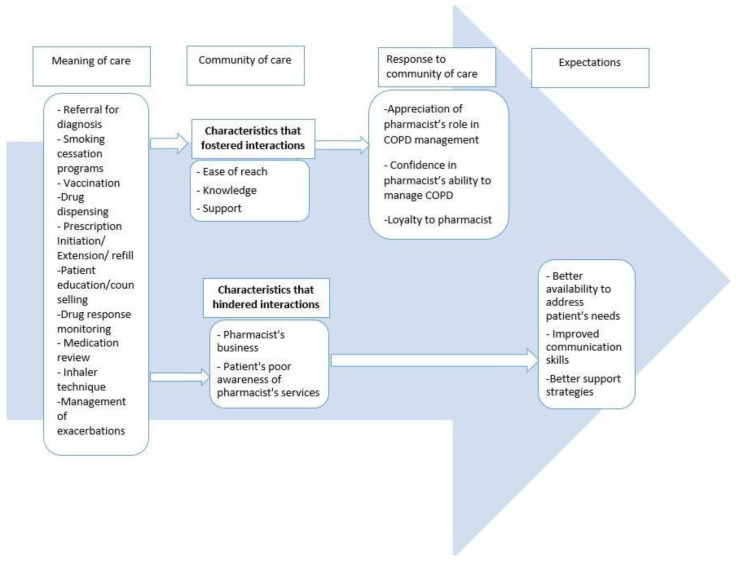
Themes, categories, and codes.

The following overarching themes emerged from the data as participants described their experience of pharmacists’ provided care: (1) meaning of care, (2) community of care, (3) participant’s response to the community of care, (4) expectations. Figure 1 shows the themes, categories, and codes.

### 3.1. Meaning of Care

This theme captures participants’ experiences of care by their pharmacists. In many cases, when asked about the healthcare providers that support their disease management, participants did not readily mention their pharmacists until further probing. Thereafter, the participants discussed specific ways through which they received care from their pharmacist, spanning the COPD diagnosis and management spectrum.

In total, 11 participants had little or no experience of discussing their symptoms prior to diagnosis with their pharmacists which was partly due to unawareness of the significance of their symptoms, as well as not seeing a pharmacist as the one helping participants better understand the implications of those symptoms, as described by the following participant:

‘I did have symptoms, but I didn’t know that it was COPD. I was always phlegmy, a lot of—wheezing, I guess, when I was laying down or resting… and tired. No energy… thinking back now, knowing now, there was all the symptoms, because, you don’t really have that intimate one-on-one conversation with your pharmacist about, “Hey, you know what?” So, I didn’t go to my pharmacist and say, “Hey, you know what, I’m wheezing today and having troubles. I bought over the counter cough syrup or halls or something like that, Vicks. I bought a humidifier…a couple of times when I did get Bronchitis, I went and got antibiotics for it. But, nobody put it together that I had COPD’.

(Participant 5, Male)

Thus, most participants did not perceive their pharmacists as instrumental in their disease diagnosis as their pharmacists were not aware of their symptoms. Additionally, it was common that participants’ COPD diagnosis was carried out during hospitalisation for other health challenges. One participant, however, described that her pharmacist was instrumental in her disease diagnosis as the pharmacist recommended a lung function test based on the patient’s respiratory symptoms:

‘Based on my symptoms and how much difficulty I would have with my asthma symptoms, and thinking that there was potentially something else going on… she did recommend that I get a lung function test done’.

(Participant 11, Female)

Other ways participants discussed that they were cared for by their pharmacist were through smoking cessation programs and vaccinations which they perceived to be helpful in their disease management. A participant said:

‘So now because a lot of the cessation aids don’t help me we discuss a lot of the mental barriers and blocks and stuff like that regarding quitting; and that’s really where my struggle. It’s not so much the physical addiction but more the mental addiction and the anxiety of ‘what am I going to do if I can’t smoke’’. And so she helps talk me through a lot of that. We’ve spent a lot of time on that consultation. It hasn’t been fully successful yet but we keep working on it’.

(Participant 11, Female)

Participants also experienced care by being able to conveniently obtain their ongoing medications, prescriptions renewed or initiated on new prescriptions for some medication, especially when they could not see their physicians. The participants deemed these services important because they improved their health or prevented their disease conditions from worsening.

‘So she can prescribe—I just think they prescribe certain medications, that you could continue for say one month, or a few days, or something like that, provided that probably you’re already on it, and you’re running out, and you can’t get to see the doctor, so they’ll provide a kind of a stopgap’.

(Participant 7, Male)

Furthermore, the participants discussed that they felt more capable of managing COPD when they received information on their medication, potential side effects, disease condition, and treatment options, or how to properly use their inhalers from the pharmacist. This was expressed by those participants whose pharmacists took their time to provide these education/counselling services, and asked questions to better understand their patients.

‘When I go in to see him and I tell him I need this or that, he’s very interactive and suggests some of—like he’ll look at my other medications and say “You know what, maybe you might not want to take that because if you take it for too long it can affect asthma, so let’s try—” Like I mean he’s always educating me which is important because I didn’t know that and I now I do. So now I have a choice to decide whether or not I change it and, of course, I’m going to change it because the last thing I need is something else to worry about, you know. That to me, that’s important’.

(Participant 1, Female)

However, in some cases, participants experienced less interaction with their pharmacists; thus, these participants did not perceive the pharmacists as a major source of information on prescribed drugs as whatever information they needed was obtained from the medication package inserts. One of those participants acknowledged it would have been easier for her if her pharmacist spoke to her instead of having to always read the inserts.

Participants also found discussions with their pharmacists about their drug response and medication review sessions beneficial as this made them more aware. The following quote is an example of a pharmacist who had detailed conversations with the participant and asked questions which were focused on medication use:

‘Well, she’s always got lots of questions. Every time I go in to pick up refills she has a lot of questions… they would ask me almost pill by pill, medication by medication how it was working and how much was I taking. They would maybe make suggestions to change the amount I was taking or the time I was taking it or something like that…She lets me know what could go wrong and is it? You know, is that happening to you? No, it isn’t. Okay, that’s good and then she goes through the benefits and is that happening to you? Yeah. Good. So, you know, she gives you both sides of the medication story’.

(Participant 8, Male)

Lastly, based on previous experiences with flare-ups and the fear of not receiving timely care during such exacerbations, some participants requested their physicians provide standing orders for antibiotics at the pharmacy. Participants found this speedy access to treatment helpful in managing their exacerbations, and the pharmacist’s involvement in this process was recognised, ‘if she thinks something on my prescriptions is wrong she’ll call him and talk to him about that’. (Participant 11, Female).

Some participants discussed that their pharmacists had to collaborate with their physicians in some instances where there was a need to initiate, change or discontinue the medication. In receiving an appropriate therapy, the following patient perceived himself as the communication link between the pharmacist and the physician:

‘Yeah, and if you say you’re experiencing this, well then you can go to the doctor and say, well, I’ve talked to the pharmacist, and they’re like, you know, suggesting this. And then they say, what do you think? So—and they say either yay or nay, and if they do say yay, they normally write you a prescription. So—but then again, too, that’s just confirmation that the pharmacist is correct. And so it’s a system—kind of a system with checks and balances, so to speak’.

(Participant 7, Male)

### 3.2. Community of Care

This theme captures participants’ experiences of how connections with their pharmacists are formed and sustained. Participants’ discussions also entail how such connections may have been hindered.

#### 3.2.1. Characteristics That Fostered Interactions

Participants identified the following to foster the interactions between a patient and a pharmacist: Ease of reach, Knowledge, Support. Overall, participants felt that their pharmacists were accessible and described them as easier to reach and talk to, compared to their physicians. Easy access to their pharmacist was convenient and less difficult than reaching the doctor, as they could reach their pharmacist, either physically or through phone calls, to address their challenges without much delay.

Beyond the perception of pharmacists’ being easier to reach when participants needed care, participants discussed that the pharmacists’ knowledge was essential in sustaining the relationships with their pharmacists. Participants also expressed they thought their pharmacist was knowledgeable when the pharmacists communicated relevant and needed information which made them feel understood. Furthermore, participants felt their pharmacist was knowledgeable when the pharmacist resolved any of their (patient) issues or provided clarity in unclear situations, ‘…She knows everything, knows her job inside and out… she pretty much knows the ins and outs, and knows what questions to ask for which patients’. (Participant 7, Male). Another participant perceived her pharmacist as knowledgeable because the pharmacist attended training programs, conferences, and seminars on respiratory care, and was able to share learnings from such programs with her.

Beyond ease of pharmacist reach and their knowledge, participants discussed that the feeling of being supported by the pharmacist was also important. Participants expressed the pharmacists supported them through consistent provision of care both in words and actions, which, according to participants, made their disease management more effective while enabling the achievement of their treatment goals. Participants acknowledged that in some instances, it involved the pharmacist doing additional work to ensure that participants received what they needed whenever needed. This made them feel as ‘individuals, not just numbers or business clients’ (Participant 8, Male). Participants also felt supported when the pharmacist considered their financial situation while recommending, or before dispensing their prescription medicines. Moreover, a participant acknowledged that her pharmacist’s concern and interest in other areas of her life which may impair her COPD management also made her feel supported and cared for by her pharmacist. On this, she said:

‘She cares on more than just a professional level… she’s always asking about how I’m doing not just about my medical issues or things like that. She’s concerned about why the symptoms are the way they are and if some other aspect of my lifestyle or health is impacting it’.

(Participant 11, Female)

The ability to communicate with their pharmacists with familiarity and humor, as friends would communicate with one another, felt good to participants, and they discussed how this helped to nurture their relationship with their pharmacists. A participant had this to say about his pharmacist:

‘The other day, she was back in the corner, and she had a mortar and pestle, or whatever, and she was mixing something up. And all I said was double, double, toil and trouble. And she says, what, are you calling me a witch? And I said, no, I’m just quoting Shakespeare. So she’s got a sense of humor, I got a sense of humor… So we do have a nice rapport back and forth. So lots of fun. It’s always a joy to go in there’.

(Participant 7, Male)

Some participants also felt supported when their pharmacists put additional effort through phone calls and home visits either to follow up on how their patients were doing or to deliver their services, especially when these patients had reduced mobility (which is common among patients with COPD). It is worth noting that while some participants described what helps in sustaining their relationships with their pharmacists, others had limited interactions and little relationship with their pharmacists.

#### 3.2.2. Characteristics That Hindered Interactions

The following categories highlighted what hindered rich patient-pharmacist interactions: pharmacists’ busyness and patients’ awareness of pharmacist’s services.

In contrast to a feeling of the pharmacist being easily accessible, some participants felt that their pharmacists were not always available for them to seek care. This was attributed to the participants’ perception of pharmacists being very busy based on recurrent experiences of being in long queues and waiting for prolonged times to pick up their prescriptions. To some of these participants, the large patient base, and sometimes, the physical setup of the pharmacy was not conducive for extensive interactions. As a result, some participants regarded the internet or drug inserts as their primary sources of drug information. Participants who had the perception of pharmacists being too busy commonly were those who sought care at larger pharmacies:

‘…There’s always a line and they’re all running around trying to get everything done especially where I go. They don’t have a lot of time to spend with each individual person so…if I’m picking them up right away I have to wait sometimes an hour or more because there’s so many people ahead of me; so I think they’re very, very busy’.

(Participant 12, Female)

Through some participants’ discussions, we noted variations in awareness of pharmacists’ services. While some participants were aware of services other than dispensing, e.g., initiation, adapting, or extending a prescription, others were not. For example, Participant 6 (Female) was unaware that her pharmacist could prescribe certain medications because ‘they [pharmacists] do not have my health records’. The same participant added that she rarely discussed her prescription medicines with her pharmacist and was unaware that her pharmacist could assess her inhaler technique. For her, the only form of care her pharmacist is associated with is the filling of prescriptions because her interactions with the pharmacist were limited to only picking up medication. This hindered better engagement and a stronger patient–pharmacist relationship.

‘I didn’t even know that pharmacists could give you a prescription without a doctor’s okay… Well, I don’t think they can prescribe a prescription for me without a doctor’s note, cause they don’t know my—they don’t have my health records, I don’t think. So, how would they know what to prescribe?’

(Participant 6, Female)

### 3.3. Participants’ Response to Community of Care

The response to the value that participants attached to the pharmacists’ care was captured by the following themes: appreciation of pharmacists’ role in disease management, confidence in pharmacist’s ability to manage COPD, and loyalty to the pharmacist.

Some participants described being grateful for the valuable care their pharmacist provided for them, as well as how the care was provided. Beyond appreciation, participants were also more confident in their pharmacists’ services based on their longstanding experience of quality healthcare. With increased confidence in their pharmacist’s practice came trust and a sense of safety that their pharmacist had their best interest in mind.

‘Since I first met her she’s just been great… she’s probably the best in the city, that’s a doctor’s opinion. If she closed her doors, I’d be in dire straits. It sounds odd when you say that about somebody you deal with. But when you find somebody that you deal with whether its medicine or buying clothes or cars and you trust them, you don’t want them to leave. Knowing that I’ve got who I have behind me in my medical situation I feel well protected’.

(Participant 9, Male)

With trust and a sense of safety came a sense of loyalty of participants to their pharmacists. Loyalty was also due to the value participants perceived they received from their pharmacists and in a lot of cases, the personal and social skills, and the friendly attitude of the pharmacists and other pharmacy staff. Loyalty is also displayed when participants access care from their pharmacy, even when it is not necessarily the closest to them or convenient to do so. It is also displayed in participants sticking with their pharmacist even with the awareness that they could obtain their prescriptions from other pharmacies at lower prices. On loyalty, a participant said:

‘I’ve been going to her for 25 years. I go out of my way because the hours that the pharmacy is open are limited compared to big commercial companies or, you know, the grocery stores that have the pharmacies in them. Honestly the reason I haven’t left is I like the personal attention that she… part of it I think is the longevity of the relationship that we’ve had, also she’s very personable and asks questions and it’s not just a service where you go in put your prescriptions in and you get your meds and out the door’.

(Participant 11, Female)

### 3.4. Expectations

This theme captures participants’ expectations of care. Based on the experience of accessing care from their current pharmacies or those they had used at some point or the other, participants had a variety of expectations of pharmacists in general. These participants’ expectations reflected a desire for continuity (for those who were well engaged by their pharmacist) or a change (for those who experienced sub-optimal care). The categories we identified here were better pharmacist’s availability to address patient’s needs, improved communication skills, and better support strategies. Participants expressed that the lack of these things had made them change their pharmacies at times during the course of their disease management. For availability, participants expressed a need for pharmacists to be more available, especially to address their needs as chronic disease patients. Improved availability entailed pharmacists being able to engage their patients as needed, despite their busy schedules. A participant described that though her pharmacist was very busy, she still found a way to attend to the needs of her patients at the pharmacy.

Some participants also discussed that respectful and friendly interactions with the pharmacist/pharmacy staff were important and preferred that in comparison to interactions that were overly formal.

They also discussed support in terms of pharmacists consistently delivering care with high quality. Pharmacists could also be supportive by being flexible in the delivery of care such as prescription drop-off and administration of vaccines, especially for individuals with reduced mobility. A participant who received vaccination in his home by his current pharmacist said his previous pharmacist would never have conducted that because in that setting, ‘everything was black and white, there were no in-betweens’ (Participant 9, Male). Some participants wished that their pharmacists were more helpful in helping them to stop smoking. Support was also seen in the light of earlier initiation of discussions on smoking cessation by the pharmacist, pharmacists being more detailed in their education and counselling services, including demonstration of inhaler technique and also providing information on programs such as pulmonary rehabilitation which the participants said could help them better manage their condition.

‘This is how you take the medication, this is what you’re supposed to do whether you rinse or gargle or whatever after. And this is how you actually do, like, inhale”. That’s how I would like it. Not just, “Here you go, this can cause this, do you understand? See you later”. I would like it if they went more into show you how to use it, explain more, give you some examples of some side effects. Because if they ask you, “Okay, do you have any side effects?” how do you know? I just might not be feeling well this day or, “Hey, I got a rash”, but I didn’t—it might affiliate with that’.

(Participant 5, Male)

‘And then I tried [name of pharmacy], because they had the low dispensing fee, one of the lowest, and they were horrible. They were just horrible. They didn’t understand what you were saying. They didn’t have your medication ready. You know, there was, like a hassle after hassle’.

(Participant 10, Female)

In communicating, however, a participant said his pharmacist should use simple language to enable him to understand medical terms, which he was unfamiliar with.

‘I don’t understand all the words that he says. I don’t understand everything. Sometimes, not often, because a lot of times—I think people, and myself included, feel less intelligent if somebody’s talking very big words or whatever, and so you’ll just agree, and even if you don’t understand them, you’ll just agree’.

(Participant 5, Male)

## 4. Discussion

This study explored the experiences of patients living with COPD of care provided by pharmacists using a qualitative descriptive methodology. Our study participants’ experience of pharmacy care varied widely based on the depth of patient–pharmacist engagements. For some participants, the pharmacist was proactively and consistently involved in patient care through the provision of medication and non-medication services. In addition to this, the pharmacist proactively collaborated, communicated, educated, engaged, connected with, and supported their patients with empathy. This made those participants value pharmacist care and regard their pharmacist as an essential member of their healthcare team. Other participants’ experiences reflect their view that pharmacists were not essential members of their healthcare team. These pharmacists had limited interactions and engagement with the study participants, which may have been linked with the pharmacists being too busy to interact with the participants and low awareness of pharmacy services in COPD care by the participants. Though the overall experiences of the participants were somewhat positive, both the challenges expressed by the participants, as well as positive experiences can serve as a ground to improve pharmacists’ role in COPD care.

Through participants’ experience of care in the timely management of exacerbations, medication dispensing by the pharmacist, and optimisation of pharmacotherapy, participants perceived their pharmacist as the medication expert and the assurer of appropriate therapy. This aligns with findings from a review by Anderson et al. that patients perceive community pharmacists as medication experts [39]. Additionally, some participants’ experience of non-medication care such as referral for diagnostic tests, health promotion, educational consultations, and smoking cessation support improved participants’ awareness and empowered participants to take responsibility for their own health. Active patient education and empowerment promote patients’ self-management, which is an important component of high-quality care, especially in chronic disease management [40]. Access to necessary information may have also helped our participants address uncertainties about their disease management or effectiveness of treatment, which are suggested to be anxiety provoking in chronic disease patients [41].

Beyond disease management activities, the way care is delivered (pharmacist’s level of patient centredness) is a major influence on the patient’s experience of pharmacy care. Effective communication (from the participants’ perspective characterised by ease of reach, knowledge, and support), provision of care with empathy, collaborative practices with other healthcare providers were elements of patient-centred care that the participants discussed. Through the pharmacists’ proactive conversations and communication (active listening, speaking, and asking relevant questions), pharmacists were able to identify and address participants’ personal challenges to smoking cessation and COPD management, as well as medical needs such as the need for confirmatory diagnosis based on patient’s symptoms. In addition to patient-centred communication which helped pharmacists identify and proffer solutions to patients’ unique needs, the experience of being supported and cared for with empathy and the humor/familiarity that characterised patient–pharmacist interactions made participants look forward to their pharmacy visits. This aligns with known findings that good communication, empathy, and support are elements of successful patient–healthcare provider relationships [20,42,43].

From participants’ experiences, the importance of pharmacist–physician collaboration is understood. Collaboration was illustrated by the physician’s standing order for antibiotics at the pharmacy, the pharmacist calling the physician to address patients’ prescription issues, and importantly, the shared decision-making process involving the patient, pharmacist, and physician in the initiation of appropriate therapy for the patient. The pharmacists’ involvement in choosing appropriate therapy for the patient is an evolutionary step in changing the narrative of pharmacists’ being just medication dispensers, which a number of studies have reported [44,45]. It is also informative that participants who discussed their pharmacist–physician collaboration and their experience of shared decision making with the pharmacist were those who regarded their pharmacists as essential members of their healthcare team. This confirms that collaboration and shared decision making are crucial components of patient-centred care [46]. Some participants, however, interpreted these collaborative efforts as a gap in care due to lack of pharmacist autonomy in making some decisions on patients’ drug therapy.

The lack of patient-centred care in some participants’ experience underscores the need for pharmacists to be deliberate in the consistent delivery of patient-centred care. Though our study did not explore how patients’ outcomes were impacted by the level of patient–pharmacist interactions, strong relationships were important to our participants, and they attributed some of their disease management successes to them. With the provision of consistent high-quality care and patient engagement, participants perceived their pharmacists as valuable. In response to this, participants had increased confidence in the pharmacists’ practice and expressed a strong sense of loyalty to their pharmacists, based on trust and a sense of safety. These findings are echoed by another study suggesting that positive customer perceptions of the pharmacist significantly influence devotion [47].

Though the overall experiences of our participants were somewhat positive, this study also highlights challenges in pharmacy care of patients with COPD, which if addressed can improve patients’ experience of care. Participants’ lack of awareness of pharmacists’ scope of practice reflected in the perception that the pharmacist does not have access to patient’s health records and may not have enough information to be able to make some clinical decisions such as prescribing medication. This observation suggests limited patients’ understanding of pharmacists’ functions which can be addressed through communication. Though pharmacists may lack access to patient records in other jurisdictions, this is not the case in Alberta where this study was performed, as Netcare (provincial health electronic records) allows healthcare professionals (pharmacists included) have access to patients’ information. Participants perceived the pharmacists as being too busy for extensive interactions based on experiences of long queues and long wait times at the pharmacy. Lack of time is a known barrier to the provision of patient-centred care by pharmacists [48]. To address the limitation of time, expansion of the roles of pharmacy technicians and other pharmacy support workforce have been made to enable pharmacists to focus more on intellectual decision making and provision of patient-centred care [49,50]. Other methods through which lack of time may be addressed include more than one pharmacist working at a time and, if allowed by the law, having a technician checking the prescriptions filled by other technicians, as against the pharmacist’s [51]. The lack of awareness of pharmacy services and the participants’ perception of pharmacists as being too busy may have limited some participants’ expectations of the pharmacist, and this aligns with existing evidence [44,47,48,52]. It is therefore important to put more effort into increasing the public’s awareness of pharmacy services to improve care and patients’ experiences of care [42,52]. It also highlights the inconsistency of pharmacy practice—many patients have never seen their pharmacist provide care.

Inadequate information/education was experienced by participants when the pharmacist’s provision of medication information was impaired either by time limitations or mode of communication. Though it may be more convenient or less time consuming for the pharmacists to direct patients to medication package inserts for information, there are some barriers that limit the effectiveness of using medication package inserts alone to educate patients about their medication. These include poor health literacy, reading difficulties by the elderly, and the use of technical language in the drug inserts which patients may not understand [53]. To address the challenge of inadequate information/education, verbal (in simple and understandable language) and written communication, with the use of visual aids/demonstrations (where appropriate), should be used concurrently to educate patients on their medication [54].

Raising awareness and promoting the prevention of COPD is an important gap in care which could be addressed by pharmacists. Based on the study participants’ experiences, there is a lack of awareness about the association between COPD symptoms, smoking (a major risk factor), and COPD, which may have contributed to participants’ late disease diagnosis. Poor disease awareness and late diagnosis are documented challenges in COPD management [5,6,55,56]. It is therefore imperative that pharmacists proactively ask their patients questions on their risk factors and symptoms, listen to these patients, educate them, and answer any questions the patients may have with empathy to improve patients’ awareness of COPD, which may lead to earlier disease diagnosis and management. Furthermore, to support patients in quitting smoking, psychological factors such as anxiety and depression should be addressed through behavioral therapy and individual counselling [55,57]. A participant’s submission, which aligns with published evidence, suggests that smoking cessation in patients with COPD may be more challenging due to these mental barriers [55]. What is important is that pharmacists should not wait to be asked by the patient, who may not know about preventive measures or what care could be provided by their pharmacist and other members of the healthcare team.

Patients’ experience of patient-centred care may be linked with their perception of the pharmacist as an essential member of their healthcare team or otherwise. Patients’ experience of pharmacy care may be enhanced if the challenges we identified are addressed. With the evolution of pharmacists’ roles and the unique needs of patients with COPD, it is essential that pharmacy care is consistently patient-centred.

### 4.1. Limitations

Our study has the following limitations. Study participants were sampled using the convenience sampling approach. Thus, the study sample may not fully represent the wide range of patients’ experiences and potentially underrepresent views of patients with limited or negative experiences. We did not target diverse regions and ethnic backgrounds or races, which might also affect the breadth of experiences with healthcare overall [58]. The exclusion of participants who could not speak English may have also led to the inability to capture the experiences of non-English speakers. Furthermore, considering that participants were recruited either through the pulmonary rehabilitation program or community pharmacies, there may be other classes of patients outside these settings with differing experiences. Another limitation is that our study participants were diagnosed in the past, which might affect their experiences with pharmacists as pharmacy care has changed more recently. Additionally, participants responded to our questions based on what they could recall, and considering that majority of them had lived with COPD for over 5 years, it is possible other relevant information may have been excluded. Additionally, most of them did not have the experience of their pharmacists being involved in their diagnosis, which we were interested in. Furthermore, all participants were recruited from an urban area, leaving out those in rural areas whose experiences may have been different based on previous findings that pharmacy practices may differ between urban and rural communities [59]. Our interview guide was also not pilot-tested prior to the commencement of the study. Participants did not have an opportunity to go through the transcripts to validate what was said during the interviews, neither did the participants go through the analysed data to provide their feedback on the appropriateness of the codes in capturing their experiences. Last, time restrictions and the current global pandemic were factors that affected the sample size and collection of data.

### 4.2. Implications for Research and Practice

Based on participant experiences, the following aspects of pharmacist-provided care can be considered when providing care to patients with COPD:

1. Patients appreciate and anticipate meaningful interaction with their pharmacists about their overall health, the use of medications, managing side effects, and assistance with smoking cessation;
2. Patients appreciate pharmacists being involved in prevention and timely management of exacerbations, e.g., pharmacist prescribing, standing orders for antibiotics;
3. Patients value pharmacists being an active collaborator with other healthcare providers, including the patient’s physician (family or specialist), e.g., in addressing prescription errors, patients’ plan of care, and in the initiation of appropriate therapy;
4. Patients value pharmacists connecting patients with resources, e.g., rehab programs;
5. Patients appreciate pharmacists’ knowledge of them, i.e., recent hospitalisations, medication use that might signal an underlying condition (COPD), and other challenges (personal or otherwise) which may impair COPD management;
6. Patients identified the importance of having knowledge of COPD, assessment of COPD symptoms and risk factors pre-disease diagnosis, and early disease identification.

In addition, it is important to note that all of these aspects of patient care require proactive action by the patient’s pharmacist and active collaboration with other healthcare team members.

Future research should explore the impact of pharmacist care of COPD on patient outcomes. As the experiences of rural patients may differ from those in urban areas, these patients’ experiences may also be explored. To understand current pharmacists’ practices with regard to early disease identification, it may be important to conduct interviews for recently diagnosed patients, as their experiences may differ from our study participants who have lived with COPD for seven years on average. Additionally, the area of collaborative practice with other healthcare providers and the role of the pharmacist on the team should be further informed.

## 5. Conclusions

Pharmacy care is important in successful COPD management. In improving pharmacy care in COPD, the experiences of patients are crucial. For patients with COPD, the following aspects of care can be important: education, communication and connection, empathy, and consistent support in disease management such as timely control of exacerbations. Our findings also indicate a need for patients’ early awareness of risk factors and symptoms of COPD, support in early disease identification, and the provision of patient-centred care based on patients’ individual needs in disease management. Lastly, the awareness of patients and other healthcare providers of pharmacy services may influence the utilisation of such services, thus affecting their overall experience of care.

## Figures and Tables

**Table 1:** Example of coding and categorising the theme ‘community of care’.

Meaning Unit	Condensed Unit	Code	Category	Theme
It’s really easy, like I go to the pharmacy and so it’s really easy for me to get any of the information I need.	Easy to reach	Ease of reach	Characteristics fostering interactions	Community of care
…I can’t get an appointment with the doctor until the next week, well then I can go to my pharmacist…	Short wait time			
it’s easier to talk to your pharmacist, because a doctor, you got to wait, and then go in.	Easier access			
She was so proud of him and she would encourage him I’m proud of you	Encouraging	Support		
She’ll phone me and ask how I’m feeling.	Follow up			
Just caring, concerned, very nice lady	Empathy			
Yeah just I would go in and whine and she would tell me what to change or do and how often.	Shoulder to lean on			
There might be other pharmacists that are not quite as busy that would have more time to interact.	Busy to interact	Pharmacists busyness	Characteristics hindering interactions	
Do you know, they’re so crazy busy there. How can they—they’re just so busy.	No time			
They don’t have a lot of time to spend with each individual person so… I don’t know.	No time			
I didn’t even know that pharmacists could give you a prescription without a doctor’s okay.	Don’t know	Participants’ awareness of pharmacy services		

**Table 2:** Overview of participant’s characteristics.

	Gender	Age (Years)	Years of Living with COPD	Site of Recruitment	Smoking Status	Frequency of Pharmacy Visit
1	Female	64	7	PRC	Former Smoker	2 to 4 visits a month
2	Male	61	8	PRC	Former Smoker	>5 times per month
3	Female	76	6	PRC	Former Smoker	2 to 4 visits a month
4	Male	75	4	PRC	Former Smoker	Less than every 3 months
5	Male	51	2	PRC	Former Smoker	2 to 4 visits a month
6	Female	85	8	PRC	Former Smoker	Once in three months
7	Male	72	5	CP	Former Smoker	Monthly
8	Male	77	22	CP	Former smoker	Monthly
9	Male	75	12	CP	Former Smoker	Monthly
10	Female	66	6	CP	Current smoker	Monthly
11	Female	46	4	CP	Current smoker	2 to 4 visits a month
12	Female	67	10	CP	Current smoker	Monthly

PRC: pulmonary rehabilitation centre, CP: community pharmacy.

## Data Availability

The data that support the findings are available from the corresponding author upon reasonable request.

## Appendix A. Interview Guide

1. How were you diagnosed with COPD and what role did your pharmacist play in your diagnosis?
2. Who helps you manage your COPD?
3. Tell me about your experiences with your pharmacists’ provided care.
4. What is the pharmacist role in caring for you?
5. Imagine that your pharmacist can do more, what would help you to manage your COPD?

Prompt used include:

1. Can you tell me more about that?
2. How did that make you feel?
3. Could you give me an example?
4. Why was that important to you?

## Appendix B. Participants’ Demographic Questionnaire

1. Gender: Male ____ Female ______ Prefer not to say _______
2. Year of birth: ______?
3. What year were you diagnosed with COPD?
4. How often do you visit a community pharmacy to access health services?
  □ More than once a month
  □ Monthly
  □ Once in two months
  □ Once in three months
  □ Less than every three months
  □ Never
5. In the past year (past 12 months), how many times have you experienced a COPD flare-up which required either additional medications (e.g., antibiotic), a visit to the emergency room, or hospitalization?
  □ 0
  □ 1
  □ 2
  □ 3
  □ Other: _____
6. Do you have any of the following health conditions? Please select all that apply.
  □ Diabetes
  □ High blood pressure
  □ Lung cancer
  □ Sleep apnea
  □ Heart disease
  □ Asthma
  □ Musculoskeletal disorder
  □ Other ________________________________________________________

## Appendix C. COREQ Checklist

	**Item No**	**Guide Guides/Description**	**Location in Manuscript/Reported on Page No**
Domain 1: Research team and reflexivity			
Personal Characteristics			
Interviewer/facilitator	1	Which author/s conducted the interview or focus group? TM and OI conducted interviews 1 to 4 together. MQ and OI conducted interviews 7 to 9 together while OI conducted interviews 5,6,10 to 12 alone.	Methods-3
Credentials	2	What were the researcher’s credentials? OI-BPharm MSc candidate MQ-RD MSc PhD TM-BS Pharm, PhD	Title page
Occupation	3	What was their occupation at the time of the study? OI-Master’s student MQ-Qualitative researcher TM-Assistant Professor	Methods-5
Gender	4	Was the researcher male or female? Females	Methods-5
Experience and training	5	What experience or training did the researcher have? OI-Took a graduate study course on qualitative research MQ-Over 11 years of experience as a qualitative researcher TM—moderate level of experience with qualitative research; formal training in qualitative research	Methods-5
Relationship with participants			
Relationship established	6	Was a relationship established prior to study commencement? No	
Participant knowledge of the interviewer	7	What did the participants know about the researcher? e.g., personal goals, reasons for doing the research Participants were briefed on the purpose of the study. Participants also reviewed the study information sheet before they gave written informed consent to be involved in the study.	Methods-3
Interviewer characteristics	8	What characteristics were reported about the inter viewer/facilitator? e.g., Bias, assumptions, reasons and interests in the research topic OI and TM acknowledged to be non-practicing pharmacists with interests in improving pharmacy care of COPD patients	Methods-5
Domain 2: Study design			
Theoretical framework			
Methodological orientation and Theory	9	What methodological orientation was stated to underpin the study? e.g., grounded theory, discourse analysis, ethnography, phenomenology, content analysis Qualitative descriptive methodology with qualitative content analysis	Methods-2
Participant selection			
Sampling	10	How were participants selected? e.g., purposive, convenience, consecutive, snowball Convenience	Methods-3
Method of approach	11	How were participants approached? e.g., face-to-face, telephone, mail, email Recruitment involved use of posters and face-to-face invitation	Methods-3 to 4
Sample size	12	How many participants were in the study? 12	Methods-3
Non-participation	13	How many people refused to participate or dropped out? Reasons? Two. One person declined to participate in the interview and another individual could not be interviewed due to the global pandemic.	Methods-3
Setting			
Setting of data collection	14	Where was the data collected? e.g., home, clinic, workplace Majority of the participants were interviewed at home. Other settings for data collection were: a meeting room at the University of Alberta and a pulmonary rehabilitation centre.	Methods-3
Presence of non-participants	15	Was anyone else present besides the participants and researchers? A non-participant (participant’s spouse) was present during one of the interviews	Methods-3
Description of sample	16	What are the important characteristics of the sample? e.g., demographic data, date Interviews were conducted from 1 October 2019 to 8 January 2020. Twelve participants- six females and six males. Their ages ranged from 46 to 85 years and they had been living with COPD between two to 22 years.	Methods-3 Results-5
Data collection			
Interview guide	17	Were questions, prompts, guides provided by the authors? Was it pilot tested? Interviews were semi-structured, using a guide which is attached as an appendix. The interview guide was iterated during the data collection process to enrich the collected data.	Methods-3
Repeat interviews	18	Were repeat interviews carried out? If yes, how many? No	
Audio/visual recording	19	Did the research use audio or visual recording to collect the data? All interviews were audio-recorded and transcribed	Methods-3
Field notes	20	Were field notes made during and/or after the interview or focus group? Field notes were made during and after the interviews.	Methods-3
Duration	21	What was the duration of the inter views or focus group? The semi-structured interviews ranged from 20 to 114 min.	Methods-3
Data saturation	22	Was data saturation discussed? In the methods section, we discussed data saturation was reached by the 12th interview.	Methods-3
Transcripts returned	23	Were transcripts returned to participants for comment and/or correction No	
Domain 3: analysis and findings			
Data analysis			
Number of data coders	24	How many data coders coded the data? At the start, OI and MQ independently coded a transcript and discussed consistency of the codes and the coding process. Thereafter, OI coded all the transcripts, with supervision and feedback by MQ.	Methods-4
Description of the coding tree	25	Did authors provide a description of the coding tree? Yes	Methods-4
Derivation of themes	26	Were themes identified in advance or derived from the data? Themes were derived from the data	Methods-4
Software	27	What software, if applicable, was used to manage the data? NVivo 12 software	Methods-4
Participant checking	28	Did participants provide feedback on the findings? No	
Reporting			
Quotations presented	29	Were participant quotations presented to illustrate the themes/findings? Was each quotation identified? e.g., participant number Comments were supported with direct quotes from participants who were anonymised by participant number and sex.	Results-6 to 13
Data and findings consistent	30	Was there consistency between the data presented and the findings? Yes	
Clarity of major themes	31	Were major themes clearly presented in the findings? Yes	
Clarity of minor themes	32	Is there a description of diverse cases or discussion of minor themes? No	
